# Two-decade dialogue between artificial intelligence and osteoporosis: research trajectories and frontier projections under bibliometric and visual analysis

**DOI:** 10.3389/fmed.2025.1606361

**Published:** 2025-11-03

**Authors:** Yun Deng, Xingyu Chen, Na Yao, Chunmei Geng, Changfei Yuan, Qigang Chen, Zhen Shen

**Affiliations:** ^1^The Third Affiliated Hospital of Yunnan University of Chinese Medicine, Kunming, China; ^2^Department of Rehabilitation, Kunming Municipal Hospital of Traditional Chinese Medicine, The Third Affiliated Hospital of Yunnan University of Chinese Medicine, Kunming, China

**Keywords:** artificial intelligence, osteoporosis, bibliometric analysis, CiteSpace, VOSviewer, R package “Bibliometrix”

## Abstract

**Purpose:**

This study systematically evaluated the intellectual progress in artificial intelligence (AI)-driven osteoporosis research between 2004 and 2024 by employing scientometric and visualization techniques. Through mapping knowledge domains and identifying emerging trends, it offered actionable recommendations and strategic insights to guide future scholarly endeavors.

**Methods:**

We queried the Web of Science Core Collection for English-language articles and reviews published between January 1, 2004, and November 30, 2024, using search terms including “osteoporosis,” “deep learning,” “convolutional neural networks,” and “artificial intelligence.” Bibliometric data were processed via VOSviewer (v1.6.20), CiteSpace (v6.3.R1), Scimago Graphica (v 1.0.46), and the R package Bibliometrix to quantify annual publication trends, assess national/institutional contributions, evaluate journal/author impact metrics, and map keyword co-occurrence and burst dynamics.

**Results:**

The bibliometric analysis identified 408 publications (343 articles, 65 reviews) from 2004 to 2024, with a marked increase in output observed in 2024. China and the United States dominated scholarly productivity and citation impact. Leading institutions included the Technical University of Munich and Seoul National University, while *Osteoporosis International* emerged as the most influential journal. Prolific authors such as Thomas Baum demonstrated significant academic leadership. Keyword co-occurrence analysis revealed deep learning, artificial intelligence, and diagnosis as core research frontiers, signaling future technological and clinical priorities.

**Conclusion:**

This study represents the first comprehensive bibliometric analysis of research on artificial intelligence in the field of osteoporosis. It not only outlines the field’s development trajectory and emerging frontiers but also highlights the research focus on AI technologies, particularly deep learning. Furthermore, it emphasizes critical challenges in clinical translation, such as algorithm optimization, model interpretability, and ethical privacy concerns. By systematically identifying key contributors, collaborative networks, and evolving research fronts, this study provides a foundational roadmap for the field. It offers strategic priorities for researchers to address methodological gaps, serves as a reference for clinicians to understand the evolving technological toolkit, and provides a basis for policymakers to promote interdisciplinary collaboration.

## 1 Introduction

Osteoporosis, a metabolic bone disorder marked by imbalanced bone remodeling (excessive resorption over formation), manifests as reduced bone mass, microarchitectural deterioration, and diminished biomechanical integrity. These pathophysiological alterations elevate fracture susceptibility, defining osteoporosis as a progressive age-associated disease (1). With the global aging population, the incidence of osteoporosis is on the rise annually, while the treatment rate is on the decline. The prevalence is higher in women than in men, particularly among postmenopausal women (2–4). According to the latest research projections, the global prevalence of osteoporosis is expected to reach a staggering 263.2 million cases between 2030 and 2034, comprising approximately 154.4 million female patients and 108.8 million male patients. Furthermore, the disease burden attributable to osteoporosis, measured by disability-adjusted life years (DALYs), is projected to reach 128.7 million during the same period, with females accounting for 78.4 million and males for 50.3 million DALYs (5). These data reveal not only the extensive global impact of osteoporosis but also highlight persistent gender disparities and a worsening public health crisis. Consequently, the prevention and treatment of osteoporosis represent an urgent global health challenge. A major difficulty in managing this disease lies in its insidious onset, as it is often asymptomatic until the first fracture occurs. This silent progression makes timely screening and early intervention critically important, as they constitute the key window for slowing disease progression and preventing devastating fractures and their associated complications.

The rapid development of Artificial Intelligence (AI), particularly deep learning and Convolutional Neural Networks (CNNs), has become a transformative force in the medical field, offering novel solutions to long-standing clinical challenges (6, 7). In medical imaging—a cornerstone of osteoporosis diagnosis—AI algorithms have demonstrated remarkable capabilities: automating image analysis, improving diagnostic accuracy, and identifying subtle patterns beyond human perception. This capability is crucial for osteoporosis, where research over the past two decades has focused on developing automated systems using X-rays, CT, and MRI for opportunistic screening and diagnosis (8, 9). Significant progress has been made in automating bone mineral density assessment and detecting osteoporotic vertebral fractures from routine clinical scans. These AI-driven tools not only exhibit diagnostic performance comparable to experts in specific tasks but also hold immense potential for standardizing diagnostic processes, enhancing early detection rates, and improving healthcare accessibility—particularly in resource-limited settings. This evolution marks a shift toward a new era of data-driven, precise, and efficient osteoporosis management.

However, the integration of artificial intelligence (AI) into routine osteoporosis care faces numerous domain-specific challenges that extend beyond technical performance. Although data heterogeneity and imaging variability are significant concerns, they represent only one facet of the problem. First, many studies are hampered by methodological limitations, including the use of small, retrospective, and single-center datasets, which raises concerns about overfitting and limits the generalizability of the findings (10, 11). Second, the “black-box” nature of many sophisticated AI models poses a critical barrier to clinical adoption. Clinicians must trust and understand the rationale behind an AI’s prediction of fracture risk, and the lack of model explainability (XAI) hinders this trust and accountability (12, 13). Furthermore, achieving seamless integration with existing clinical workflows remains a major obstacle in the practical application of AI (14). Finally, pressing ethical considerations regarding the privacy of sensitive patient data and the potential for algorithmic bias to exacerbate existing disparities in healthcare access and outcomes must be proactively addressed before widespread deployment can be considered (15). These multifaceted challenges underscore the complex transition from algorithm development to genuine clinical utility in the field of osteoporosis.

Despite the growing body of literature and the critical challenges outlined above, a comprehensive bibliometric analysis that systematically maps and synthesizes the intellectual landscape of AI applications in osteoporosis is currently lacking. Such a study is crucial for consolidating knowledge, identifying evolutionary patterns, and guiding future research directions in this rapidly developing field. To address this gap, we employed bibliometric methods, which are uniquely suited to provide a quantitative and systematic mapping of large research landscapes, thereby objectively identifying key trends, contributors, and intellectual foundations (16). We employed quantitative bibliometric methods, utilizing software such as VOSviewer and CiteSpace, to conduct a longitudinal analysis of the AI in osteoporosis research domain. Specifically, this study aims to answer the following research questions: What are the seminal publications and major intellectual turning points that have shaped the development of the field? What are the current research frontiers and emerging hotspots, and how have they evolved over time? Which countries, institutions, and authors have made the most significant contributions, and what is the nature of their collaborative networks? By addressing these questions, this study seeks to provide an objective and comprehensive overview and delineate strategic priorities for advancing AI applications in osteoporosis diagnostics and management.

Research Team Background: The authors of this study are clinicians and researchers from [Kunming Hospital of Traditional Chinese Medicine / The Third Affiliated Hospital of Yunnan University of Chinese Medicine] with extensive clinical experience in diagnosing and managing osteoporosis. Our research group has a sustained interest in exploring innovative technologies, particularly the application of artificial intelligence in medical imaging, to improve the precision and efficiency of orthopedic diagnostics. This bibliometric analysis was undertaken to systematically map the knowledge landscape of this rapidly evolving field, leveraging our clinical insights and research focus.

## 2 Materials and methods

### 2.1 Literature screening and inclusion protocol

The Web of Science Core Collection (WoS-CC) was selected as the sole data source for this bibliometric analysis. This decision was based on the following methodological advantages: (1) WoS-CC provides high-quality, standardized, and rigorously curated citation data; (2) It ensures methodological reproducibility; and (3) Focusing on a single high-quality database enhances data consistency. While this approach strengthens internal validity and consistency, we acknowledge that it may not capture all relevant literature, potentially introducing database selection bias.

We collected literature on December 10, 2024, from the Science Citation Index Expanded (SCI-E) and Social Science Citation Index (SSCI). The Search query design: #1: TS = (osteoporosis OR osteopenia OR osteoporotic OR “bone loss*” OR “low bone mass” OR “low bone density”) OR AB = (osteoporosis OR osteopenia OR osteoporotic OR “bone loss*” OR “low bone mass” OR “low bone density”), #2: (TS = “deep learning” OR TS = “convolutional neural network*” OR TS = “Artificial Intelligence”), final = #1 AND #2. After the initial search, a total of 470 documents were retrieved; research in this area was first seen in 1999, but only 1 article was published between 1999 and 2004. We decided to set the search period from January 1, 2004 to November 30, 2024 due to the low number of publications and the poor visualization of the literature during this period. The types of literature were limited to “article” and “review.” The language was set to English.

Titles and abstracts underwent dual-phase screening against predefined eligibility criteria: (1) Studies must employ AI methodologies (e.g., deep learning, machine learning, automated systems). (2) Relevant studies involving osteoporosis, including: (i) Direct associations with osteoporosis pathogenesis or diagnostics. (ii) Studies targeting the clinical features of osteoporosis. (iii) Explorations of osteoporosis comorbidity mechanisms.

**Figure 1:**
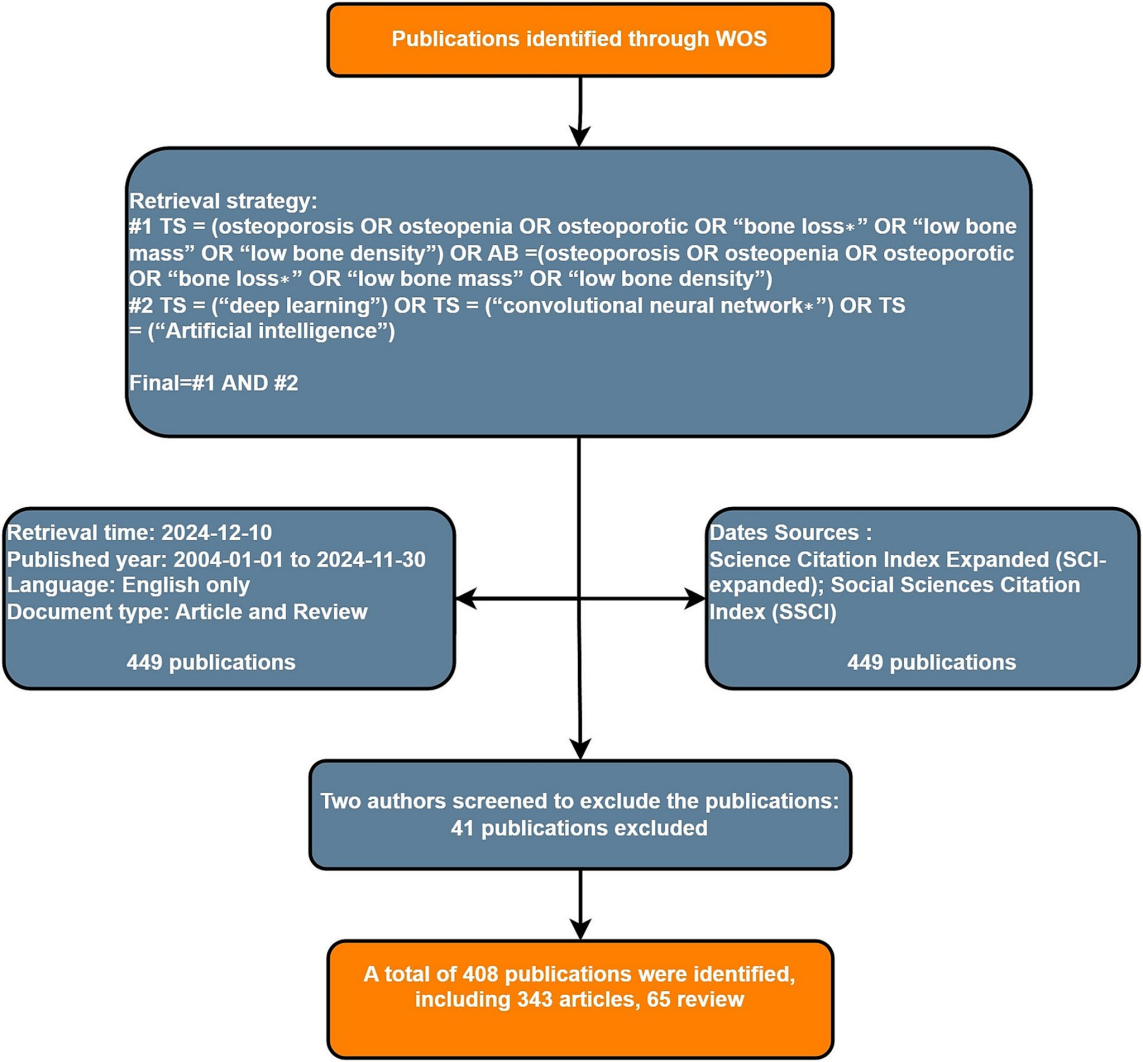
Literature screening process. The flowchart illustrates the identification, screening, eligibility assessment, and inclusion of publications for the bibliometric analysis.

Following the initial search, a rigorous data cleaning procedure was implemented. Duplicate records were identified and removed by systematically comparing titles, author lists, and Digital Object Identifiers (DOIs). The decision to include only English-language literature was based on the fact that the Web of Science Core Collection predominantly indexes high-impact international journals published in English, which represent the core discourse in this rapidly evolving and globally collaborative field. This approach ensures consistency and reproducibility in the analysis. However, we acknowledge that it may introduce a language bias by excluding relevant studies published in other languages. Similarly, the exclusion of grey literature and preprints was a deliberate choice to focus the analysis on peer-reviewed, established knowledge, albeit at the potential cost of omitting very recent findings or valuable contributions from other publication formats. A total of 408 documents were finally included for bibliometric analysis, including 343 articles and 65 reviews. The flow chart of the screening process is detailed in Figure 1.

### 2.2 Data analysis

To comprehensively assess the research landscape of artificial intelligence in the field of osteoporosis, this study employed multiple bibliometric tools for cross-verification and complementary analysis. VOSviewer (v 1.6.20) was used to construct visual maps of collaboration networks and keyword co-occurrence; CiteSpace (v 6.3.R1) leveraged its strengths to identify research fronts and delineate the temporal evolution of the field; while the Bibliometrix R package was responsible for core data preprocessing and descriptive statistics to ensure analytical reproducibility. This multi-tool integration strategy aims to reveal the complete research picture from different dimensions, with the selection based on their unique functions to address specific analytical needs (17). Supplementary tools including Microsoft Excel (2021) and Scimago Graphica (v 1.0.46) were utilized for basic data management, publication trend charting, and geospatial mapping of international collaborations, respectively.

A total of 408 documents were screened for this study, and Microsoft Office Excel (2021) was employed to manage the annual number of publications, conduct annual trend analysis, and perform linear regression on relevant variables. We utilized CiteSpace (v 6.3.R1), VOSviewer (v 1.6.20), Scimago Graphica (v 1.0.46) and the R package “Bibliometrix” to conduct scientometric analysis and visualization of data such as authors, countries/regions, institutions, and keywords from the screening results.

### 2.3 Procedures for analysis

#### 2.3.1 Microsoft Office Excel 2021 and Scimago Graphica (v 1.0.46)

We used Microsoft Office Excel 2021 to analyze and generate graphs for the annual and cumulative number of articles in this database, and also added an index analysis of the cumulative annual number of articles ( $\mathrm{Eq}:y=0.4169e^{0.2802x}\;R^{2}=0.9431$ ). Scimago Graphica (v 1.0.46), an open-access geospatial visualization tool, facilitates multidimensional data mapping. In this study, this tool was implemented to delineate inter-country/region collaborative linkages. The national collaboration network constructed via VOSviewer was exported in Geography Markup Language (GML) format and integrated into Scimago Graphica (v 1.0.46). Data attributes were systematically assigned to designated tags for generating thematic visualizations.

#### 2.3.2 VOSviewer

We saved the screened literature in tab delimited file format with full record and cited reference selected for the record. Before importing into VOSviewer, a Save As operation was performed to convert the tab delimited file encoding to UTF-8. Then the publication and collaboration maps of countries, institutions, journals, and authors were performed separately, along with the visual analysis of keyword co-occurrence, and the visual mapping of co-cited journals and authors. The node size indicates the number of publications or keyword occurrences corresponding to nodes, and the thickness of the connecting lines correlates with the strength of the collaboration between the nodes or the strength of the co-citation relationship. The inclusion thresholds (such as the minimum number of occurrences for keywords) were determined empirically. We employed an iterative approach: starting with a low threshold to capture the complete network structure, then progressively increasing the threshold until a clear and interpretable visualization was achieved, while ensuring the retention of major thematic clusters.

#### 2.3.3 R package “Bibliometrix”

The “Bibliometrix” R package, executed within the R Studio environment, requires installation prior to launching its interface. Filtered data files can then be uploaded for analytical visualization. Notably, Web of Science data must be exported in BibTeX format to maintain compatibility with Bibliometrix. We used this program to analyze national citations counts, annual growth of institutional publications, journal and author H-index values.

#### 2.3.4 CiteSpace

A plain text file was exported from Web of Science and imported into CiteSpace (v 6.3.R1). The following parameters were configured: G-index = 25, lookback year (Lby) = 5, link retention factor (Lrf) = 2.5, exponential smoothing parameter (e) = 1.0. Pathfinder and pruning slice network options were enabled to emphasize critical nodes and enhance visual clarity in the network.

The time span was from 2004 to 2024, with a time slice of 1 year. We used CiteSpace (v 6.3.R1) to conduct keyword co-occurrence analysis and burst detection analysis. The data for configuring the detection model are as follows: f(x) = αe^−αx^, α_1_/α_0_ = 2.0, α_i_/α_i − 1_ = 2.0; The number of states = 2; *γ* = 0.1; minimum duration = 2; Burst items found = 25. Additionally, we also conducted overlay of journal biplots, and the labels on the map represented relevant journals or research fields. Citation paths from citing to cited entities illustrated interdisciplinary knowledge flows. Path color saturation and thickness reflected citation intensity and temporal spans, assisting researchers in rapidly identifying stage-specific academic trends and impacts.

### 2.4 Consideration of analytical robustness

We acknowledge that bibliometric visualizations can be sensitive to parameter choices. To ensure the robustness of our findings, we conducted sensitivity analyses (e.g., by adjusting the keyword co-occurrence threshold in VOSviewer). The core network structures and major clusters remained stable across reasonable parameter ranges. All results were subjected to critical interpretation by the authors to avoid over-reliance on automated outputs.

### 2.5 Reproducibility and data availability

To facilitate research reproducibility, this study has documented the specific versions of all software tools used (e.g., VOSviewer v1.6.20, CiteSpace v6.3.R1) and reported key analytical parameters in the Methods section. The raw data supporting the findings of this study are available from the corresponding author upon reasonable request.

## 3 Results

### 3.1 Temporal dynamics of scholarly output

**Figure 2:**
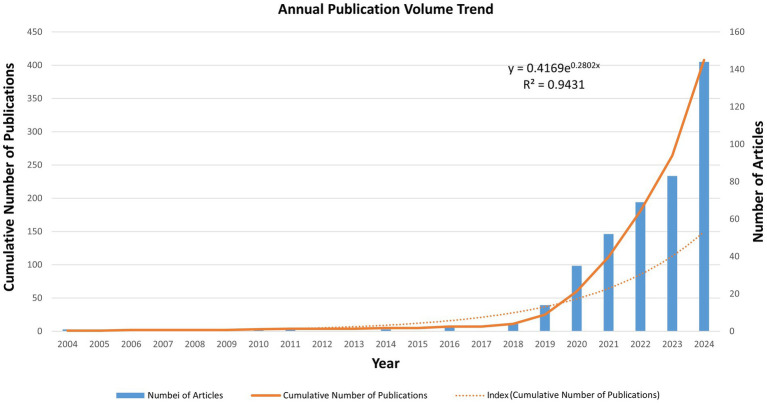
Annual publication trends from 2004 to 2024. The line graph depicts the temporal evolution of scholarly output, highlighting a notable surge in publications on artificial intelligence in osteoporosis research after 2018.

Following the screening process detailed in Figure 1, which ultimately yielded 408 documents for analysis, we first examined the annual publication trends from 2004 to 2024. The number of publications reflects, to some extent, the importance and development of particular areas by researchers. Figure 2 shows the publication trend of AI applications in osteoporosis research from 2004 to 2024. Between 2004 and 2018, annual publications did not exceed 5. From 2018 to 2023, a gradual increase was observed, followed by explosive growth in 2024, with publications reaching 144—a 73.49% increase from the prior year and a 10.3-fold rise compared to 2019 (14 publications). These trends illustrate a dramatic surge in scholarly attention in AI-driven osteoporosis research over the past 7 years, culminating in a peak in 2024.

This dramatic surge, particularly the peak observed in 2024, likely reflects the collective influence of multiple factors extending beyond purely scientific advancement. It coincides with the global proliferation and application of advanced deep learning architectures, such as Transformer-based models, within medical imaging (18–20). Furthermore, the growing policy emphasis on AI in healthcare, exemplified by the evolving regulatory frameworks for AI-based Software as a Medical Device (SaMD) from the U.S. FDA, may have stimulated accelerated R&D efforts (21, 22).

### 3.2 National contributions and institutional partnership analysis

**Figure 3:**
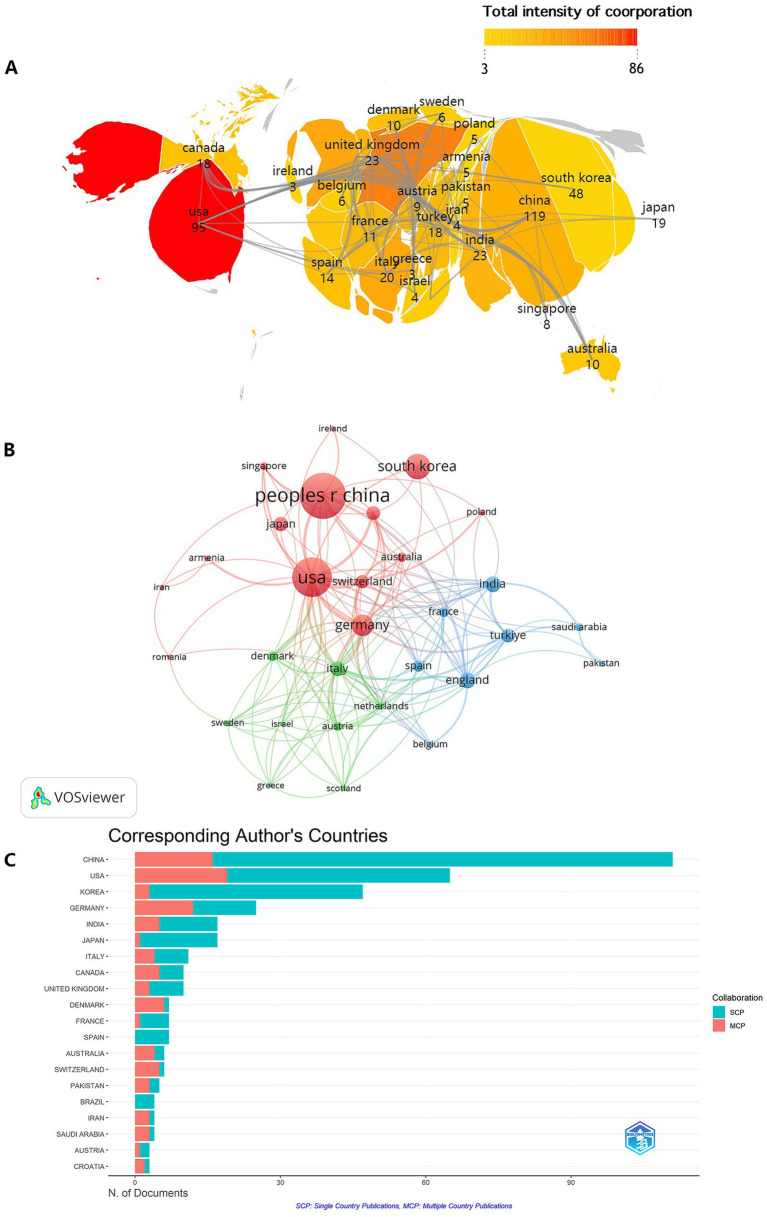
Global contribution and collaboration patterns. **(A)** A world map filled with colors indicates the volume of publications and the intensity of international collaboration across different countries. **(B)** A network visualization generated by VOSviewer displays the strength of collaborative links between countries, where node size represents publication volume and line thickness represents collaboration strength. **(C)** A bar chart shows the distribution of corresponding authors’ countries, ranked by their contributions to Single Country Publications (SCP, blue) and Multiple Country Publications (MCP, orange), revealing different national collaboration strategies.

A total of 53 countries and 903 institutions contributed to this study. According to the global geographic network map shown (Figure 3A), the top ten contributing countries are from Asia, North America and Europe, which are China, the United States, South Korea, Germany, the United Kingdom, India, Italy, Japan, Turkey and Canada. Among them, China (119 articles) and the United States (95 articles) accounted for 52.45% of the total screened publications (Table 1), highlighting their dominant influence. Of these 10 countries, 7 are developed countries, underscoring their leadership in AI-driven osteoporosis research. This not only shows that economically developed regions have abundant technological resources, superior innovation ability and huge medical markets, but also indicates that the aging population in these regions is becoming increasingly severe, and osteoporosis is a problem that is in need of urgent solution. Meanwhile, collaboration between economically advanced countries on all continents has facilitated the sharing of knowledge and technology, accelerating the progress of AI in osteoporosis research. The darker color in the global filled-color chart indicates that the country has closer research collaboration with other countries, and the chart shows that there is a more visible collaboration between the U.S. and Germany; China, the U.K., and Italy also have notably close collaboration. Country cooperation mapping via VOSviewer (Figure 3B) reveals node sizes proportional to publication volumes and line thicknesses corresponding to collaboration strength.

**Table 1:** Top 10 countries in terms of publications in the field of artificial intelligence in osteoporosis.

Rank	Country	Articles	Citations	TLS	Rank	Institution	Articles	TLS
1	China	119	1,372	32	1	Tech Univ Munich	49	60
2	USA	95	1766	95	2	Seoul Natl Univ	48	31
3	South Korea	48	889	5	3	Yonsei Univ	45	19
4	Germany	37	547	67	4	Indiana Univ Sch Med	42	17
5	United Kingdom	23	256	39	5	Univ Washington	31	20
6	India	23	190	29	6	Peking Univ	30	9
7	Italy	20	263	46	7	Chang Gung Univ	25	22
8	Japan	19	336	3	8	Korea Univ	25	16
9	Turkey	18	160	31	9	Univ Ulsan	24	5
10	Canada	18	300	21	10	Shanghai Jiao Tong Univ	22	18

The number of national citations is an important indicator of the extent to which a country’s scientific research literature is recognized by other countries or institutions, and reflects the world influence and academic status of that country’s scientific research. The United States had the highest citation count (1,766), followed by China (1,372), and then South Korea (889) and Germany (547) (Table 1).

Single country publications (SCP) quantify domestically co-authored research articles within a nation in the scientific field, which is an indicator of the extent and intensity of scientific cooperation within a country, while multiple country publications (MCP) conversely, measure internationally collaborative papers involving authors across borders, reflecting transnational research engagement. This indicator is used to evaluate the degree of participation and influence of a country in international scientific cooperation. China exhibited a significantly higher SCP than other countries, but its MCP remained relatively low compared to that of the United States (Figure 3C), suggesting limited global engagement in AI-driven osteoporosis research. China’s high SCP but relatively low MCP ratio suggests that while its research ecosystem is highly productive, it may remain somewhat insular. This pattern may stem from several factors: a large domestic market and funding priorities focused on internal challenges; potential language barriers in international collaboration; or a research culture that has historically prioritized rapid output volume. In contrast, the higher MCP ratio of the United States underscores its established role as a global research integrator, with stronger historical ties and policies conducive to international cooperation. This divergence highlights distinct strategic approaches to knowledge production, with important implications for the global dissemination and validation of research findings.

**Figure 4:**
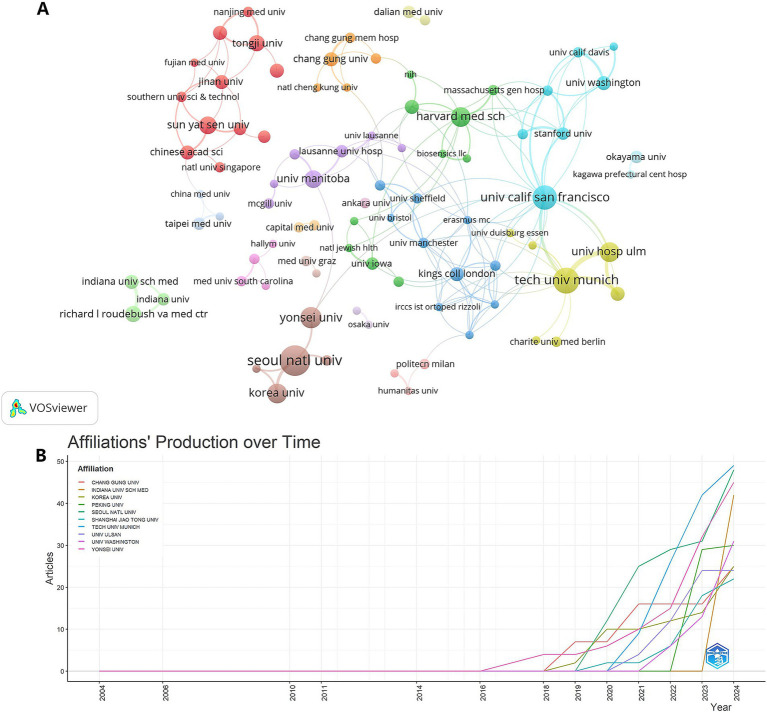
Institutional productivity and collaboration. **(A)** A network map visualizes collaborations between institutions (nodes) and their respective publication outputs (node size). **(B)** A line chart tracks the annual publication growth of the top 10 most productive institutions over time.

Total link strength (TLS) reflects the degree of connectivity and influence of a node in the overall network. A higher TLS of an institution indicates a wider range of collaborative relationships with other institutions, and also implies that it plays the role of a bridge or hub in international cooperation. Ninety-five institutions were analyzed through VOSviewer, each with more than three documents (Figure 4A). The TLS of the top five organizations in terms of posting volume is in order: Technical University of Munich (TLS = 60), Seoul National University (TLS = 31), Chang Gung University (TLS = 22), University of Washington (TLS = 20), Yonsei University (TLS = 19), Shanghai Jiao Tong University (TLS = 17) (Table 1).

Although Technical University of Munich and Seoul National University had similar publication outputs, their marked TLS difference highlights Munich’s stronger role as a collaborative hub. This non-linear correlation reflects divergent strategies: high-TLS institutions prioritize global partnerships, whereas high-output institutions may focus on independent or regional research. The role of the Technical University of Munich as a collaborative hub likely benefits from its location within the European Research Area, which actively promotes cross-border collaboration and maintains relatively unified ethical and data protocols, significantly lowering the threshold for international cooperation. In contrast, institutions such as Seoul National University may place greater emphasis on deepening domestic or regional research systems, or may be constrained by geopolitical factors, resulting in a high-output yet relatively concentrated collaborative network. This disparity implies that merely pursuing high publication volume does not equate to possessing broad global influence; proactively building and integrating into international collaboration networks is equally critical for enhancing an institution’s leadership position in globalized science.

Figure 4B illustrates the annual publication trends of the top 10 institutions. Yonsei University was the initiator in contributing to the field and has maintained consistent output. Meanwhile, Indiana University School of Medicine, a rising star, although a late entrant to the field, demonstrated rapid growth since 2023.

### 3.3 Journal impact analysis and collaborative citation networks

**Figure 5:**
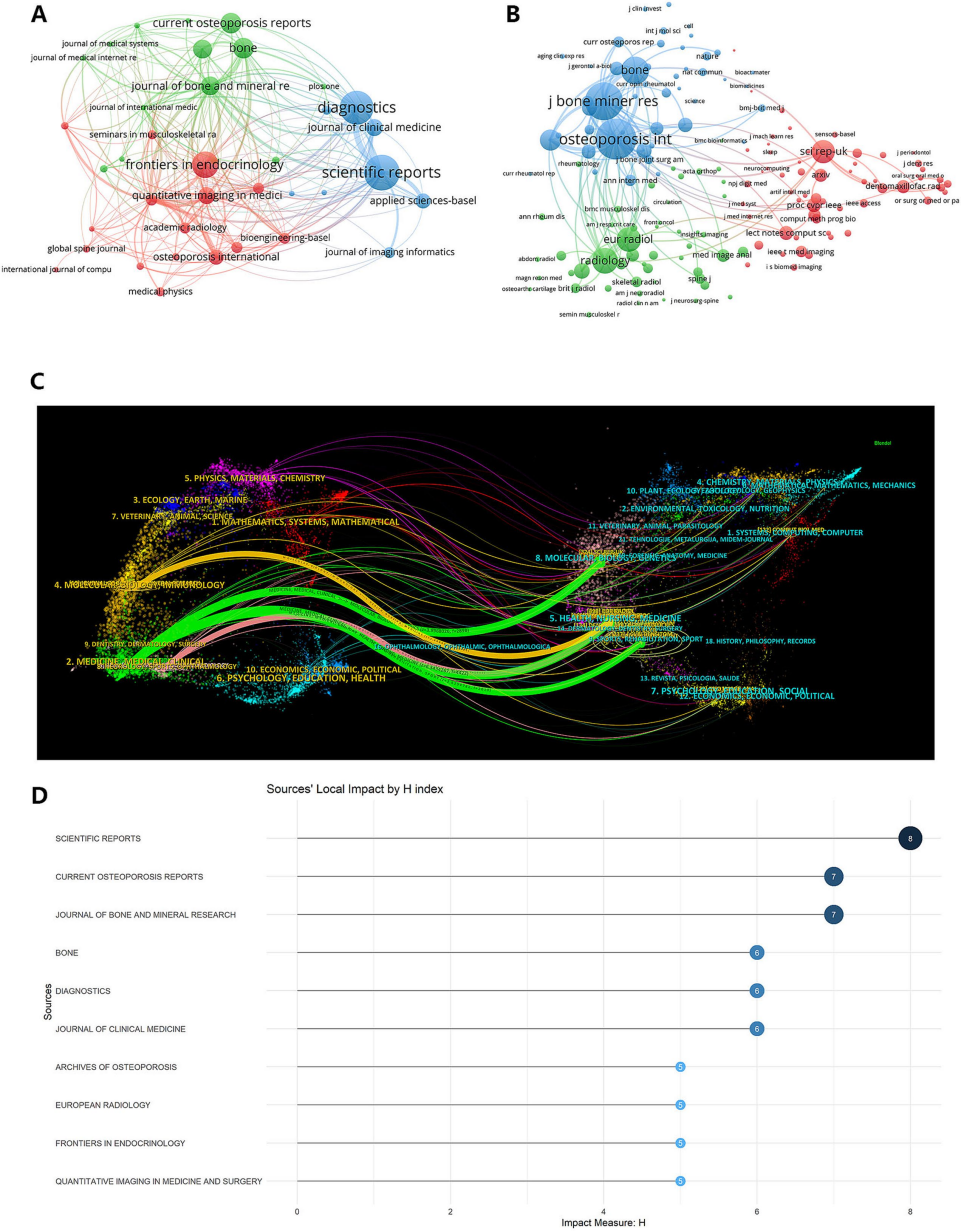
Journal influence and citation landscape. **(A)** A network map shows the co-occurrence and publication volume of active journals in the field. **(B)** A co-citation network map illustrates the relationships between journals that are frequently cited together by other papers. **(C)** A bar chart compares the H-index values of the leading journals, reflecting their combined impact and productivity. **(D)** A dual-map overlay depicts the flow of citations, with citing journals on the left and cited journals on the right, revealing interdisciplinary knowledge connections.

The dataset included 178 journals, with the top 10 journals by publication output listed in Table 2. Using VOSviewer, we visualized the journals and co-cited journals within this research field. Figure 5A displays the 39 most active journals (≥3 publications), where node sizes correspond to publication counts. Figure 5B illustrates a co-citation network of 157 journals (≥20 citations), with line thicknesses reflecting TLS values. The top five journals by TLS were *Osteoporosis Int* (TLS = 32,034), *J Bone Miner Res* (TLS = 28,624), *Bone* (TLS = 17,336), *Radiology* (TLS = 15,173) and *Eur Radiol* (TLS = 14,240). These journals closely aligned with the cited journals in Figure 5C, underscoring their authority and role as key references for researchers.

**Table 2:** Top 10 journals and co-cited journals for artificial intelligence in osteoporosis.

Rank	Journal	Counts	H-index	IF	JCR	Co-cited journal	Citations	TLS
1	Scientific Reports	19	8	3.8	Q1	Osteoporosis Int	917	32034
2	Diagnostics	18	6	3.0	Q1	J Bone Miner Res	758	28624
3	Frontiers in Endocrinology	14	5	3.9	Q2	Bone	476	17336
4	Bone	11	6	3.5	Q2	Radiology	412	15173
5	Current Osteoporosis Reports	11	7	4.2	Q1	Eur Radiol	399	14240
6	Journal of Bone and Mineral Research	10	7	5.1	Q1	Sci Rep-UK	325	12962
7	Archives of Osteoporosis	10	5	3.1	Q1	J Clin Densitom	237	11205
8	Quantitative Imaging in Medicine and Surgery	9	5	2.9	Q2	Proc Cvpr Ieee	196	5676
9	Journal of Clinical Medicine	9	6	3.0	Q2	Lect Notes Comput Sc	184	5752
10	Osteoporosis International	8	4	4.2	Q1	Arxiv	177	5226

The dual-map overlay of journal citation flows (Figure 5C) comprises citing periodicals on the left and cited periodicals on the right, with connecting lines denoting directional citation relationships. Distinct color-coded pathways revealed two predominant citation patterns: (1) Confluent Pattern (Green lines): *Osteoporosis International* exhibited dispersed citations to multiple journals, including *Sci Rep-UK*, *Eur Radiol*, and *Bone*, reflecting interdisciplinary knowledge integration. (2) Divergent Pattern (Yellow/Pink lines): Articles from *Journal of Bone and Mineral Research* and *European Spine Journal* predominantly cited *European Radiology*, demonstrating concentrated knowledge dissemination within a specialized domain. These patterns signify heterogeneous knowledge diffusion strategies in AI-osteoporosis research—confluent flows emphasize breadth, while divergent flows prioritize depth. Cluster-specific journal interactions and citation dynamics are further detailed in Figure 5C.

In journals, The H-index serves as a metric for journals academic impact and also provide scholars with directions for submitting manuscripts. Journals demonstrating the highest H-index values within this domain included the following top five: *Scientific Reports* (8), *Current Osteoporosis Reports* (7), *Journal of Bone and Mineral Research* (7), *Bone* (6), *Diagnostics* (6). H-index values showed limited variation among major journals (Figure 5D).

### 3.4 Authors and co-cited authors networks

**Figure 6:**
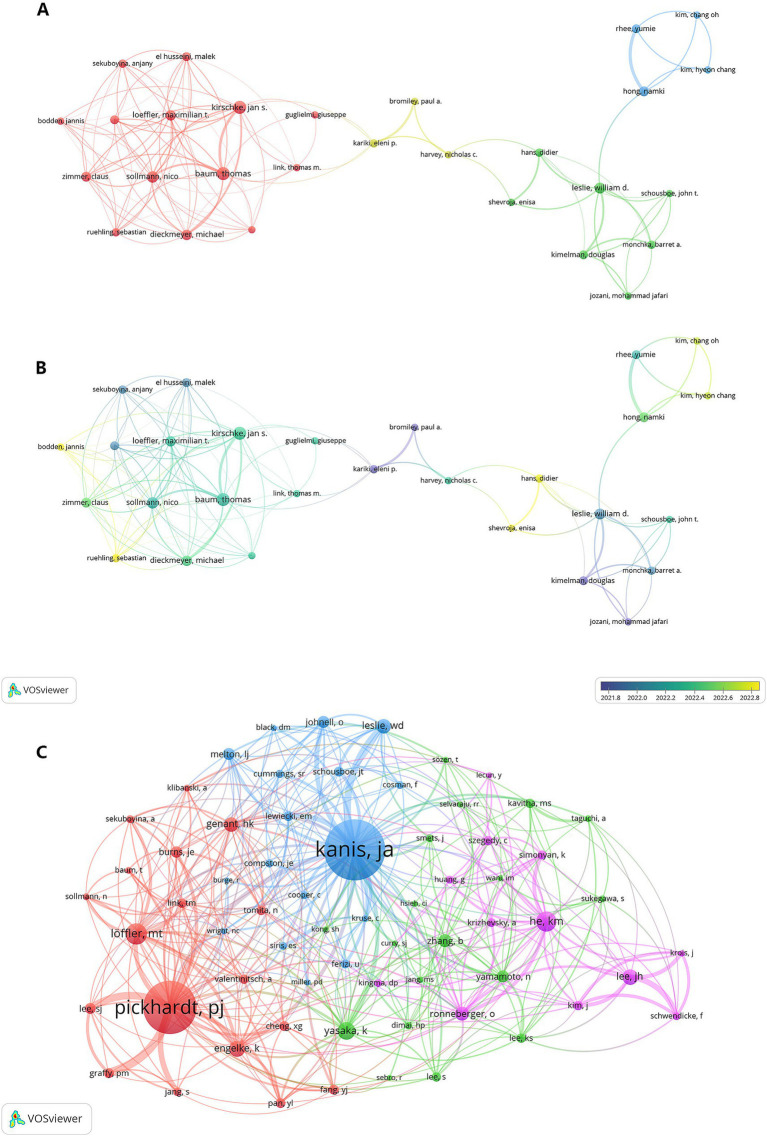
Author collaboration and influence. **(A)** A network map visualizes collaborative clusters among authors. **(B)** An overlay visualization map shows the chronological activity of authors, with blue colors indicating earlier activity and yellow colors indicating more recent activity. **(C)** The network diagram illustrates the co-citation relationships among authors.

The top 10 researchers, ranked based on publications and citation impact, are presented in Table 3. Utilizing the fractional counting method in VOSviewer, 2,431 authors were analyzed, with 120 meeting the threshold of ≥3 publications. Figures 6A,B illustrate the authors’ partnerships and active times. We can see that closer collaboration exists between authors from the same country and that systematic collaboration began emerging in 2022. The most prolific researchers—Thomas Baum, Jan S. Kirschke, and Nico Sollmann—demonstrated significant influence with 11, 11, and 9 publications, respectively. Their collaborative work in 2022 pioneered AI-driven image analysis for osteoporosis diagnosis and differentiation (23–26).

**Table 3:** Top 10 authors and co-cited authors in terms of publications.

Rank	Author	Counts	H-index	Co-cited Author	Citations	TLS
1	Baum T	11	4	Kanis JA	203	2082
2	Kirschke JS	11	5	Pickhardt PJ	178	1766
3	Sollmann N	9	6	Loeffler MT	71	776
4	Leslie WD	8	2	He KM	68	396
5	Loeffler MT	7	4	Yasaka K	60	592
6	Kacena MA	7	6	Engelke K	54	666
7	Dieckmeyer M	7	5	Lee JH	52	278
8	Pickhardt PJ	7	6	Leslie WD	49	606
9	Zimmer C	6	3	Genant HK	49	421
10	Yamamoto N	6	4	Ronneberger O	48	220

Employing VOSviewer for author co-citation analysis, Figure 6C depicts a network of 65 co-cited authors (≥20 citations each). As summarized in Table 3, prominent scholars with the highest cross-referencing frequency were: Kanis JA (co-citation = 203), Pickhardt, PJ (co-citation = 178) and Loffler, MT (co-citation = 71).

The H-index, a hybrid metric quantifying research productivity and impact, was introduced by physicist Jorge Hirsch (University of California, San Diego) in 2005. It calculates the maximum value *h* where a researcher has *h* publications each cited at least *h* times, with remaining papers cited ≤ *h* times. This index balances publication quantity (*N*) and citation quality, serving as a robust indicator of sustained scholarly influence. Positioned centrally in the network, high H-index authors are both productive researchers and key figures, with larger node sizes reflecting their enduring influence through widely cited studies that advance the field.

As shown in Table 3, Sollmann N, Kacena MA, and Pickhardt PJ have the highest H-indices. Pickhardt PJ’s most influential article was published in Radiology, demonstrated that automated AI algorithms have the potential to promote the widespread use of body CT. If proven to improve health outcomes and cost-effectiveness, opportunistic CT screening could attract healthcare systems and payers, and may even justify independent CT screening programs (27).

### 3.5 High-impact articles

Table 4 lists the 10 most cited articles on AI in osteoporosis from 2004 to 2024. The selected publications demonstrate that AI technologies primarily focus on the groundbreaking potential of AI for BMD prediction, fracture risk stratification, and treatment regimen optimization, but they also point to some core bottlenecks in clinical applications. However, these studies highlight persistent challenges in translating AI innovations into clinical practice, including model generalizability and interpretability limitations.

**Table 4:** The top 10 cited literatures in the field of artificial intelligence in osteoporosis.

Rank	Author	Title	Cited frequency	Year	Journal
1	Yasaka K	Prediction of bone mineral density from computed tomography: application of deep learning with a convolutional neural network	45	2020	Eur Radiol
2	Zhang B	Deep learning of lumbar spine X-ray for osteopenia and osteoporosis screening: A multicenter retrospective cohort study	37	2020	Bone
3	Fang YJ	Opportunistic osteoporosis screening in multi-detector CT images using deep convolutional neural networks	34	2021	Eur Radiol
4	Pan YL	Automatic opportunistic osteoporosis screening using low-dose chest computed tomography scans obtained for lung cancer screening	31	2020	Eur Radiol
5	Jang S	Opportunistic Osteoporosis Screening at Routine Abdominal and Thoracic CT: Normative L1 Trabecular Attenuation Values in More than 20,000 Adults	28	2019	Radiology
6	Yamamoto N	Deep Learning for Osteoporosis Classification Using Hip Radiographs and Patient Clinical Covariates	28	2020	Biomolecules
7	Lee KS	Evaluation of Transfer Learning with Deep Convolutional Neural Networks for Screening Osteoporosis in Dental Panoramic Radiographs	27	2020	J Clin Med
8	Valentinitsch A	Opportunistic osteoporosis screening in multi-detector CT images via local classification of textures	25	2019	Osteoporosis Int
9	Compston JE	Osteoporosis	25	2019	Lancet
10	Smets J	Machine Learning Solutions for Osteoporosis-A Review	25	2021	J Bone Miner Res

### 3.6 Keyword analysis

Keywords serve as pivotal elements in bibliometric analysis, revealing research themes and tracking field-specific hotspots and trends. Keyword co-occurrence analysis identifies latent connections among high-frequency terms, uncovering interdisciplinary intersections and informing collaborative research strategies.

**Figure 7:**
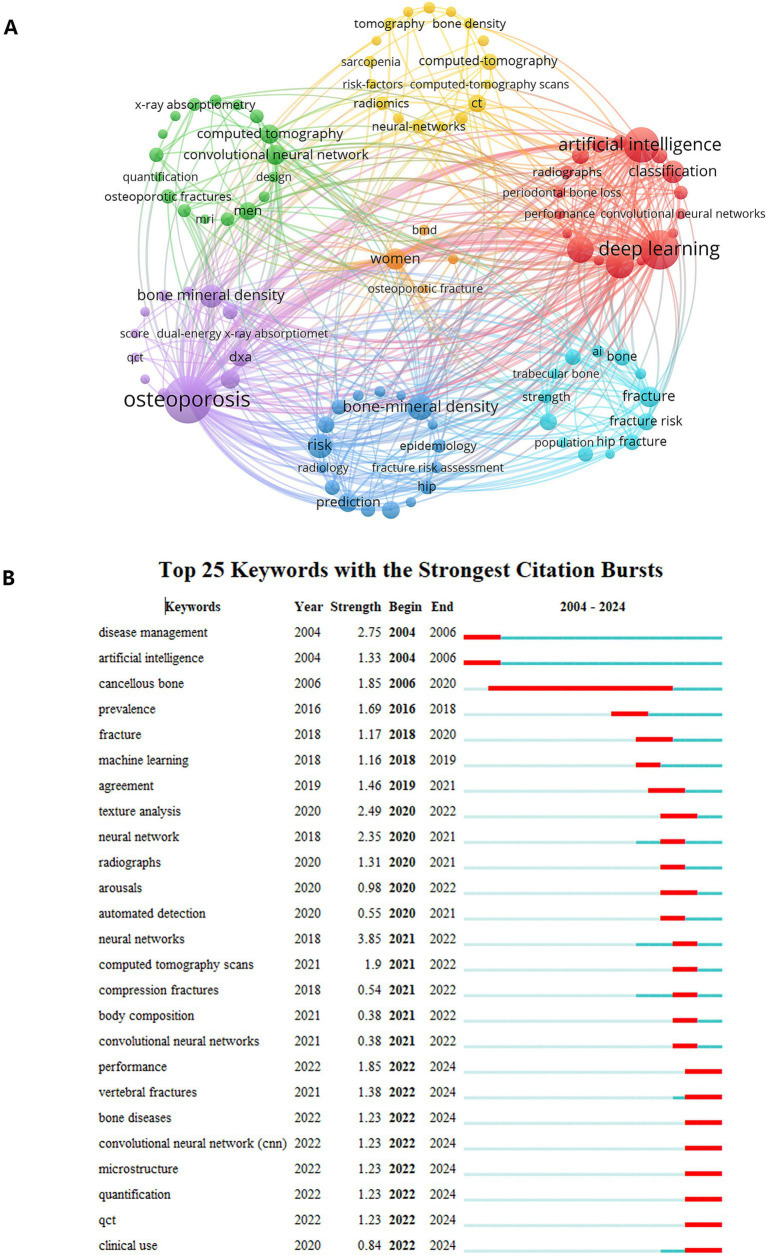
Thematic evolution and research hotspots. **(A)** A keyword co-occurrence network map identifies core research themes, where the size of the nodes corresponds to the frequency of keyword occurrence. **(B)** A bar chart lists the top 25 keywords with the strongest citation bursts, identifying the research frontiers that attracted the most significant attention over specific time periods.

Using VOSviewer, we conducted a keyword analysis and selected 106 keywords with at least 6 occurrences (Figure 7A). The top 5 keywords in the co-occurrence rankings were: osteoporosis (231 times), deep learning (149 times), artificial intelligence (115 times), bone mineral density (104 times), diagnosis (70 times). Notably, many imaging-related words appear in the keyword co-occurrences, such as CT, DXA, X-ray absorptiometry, radiomics, radiographs, underscoring the centrality of AI-enhanced imaging tools in osteoporosis diagnostics.

Table 5 lists the 20 most frequent terms in the field. It can be seen that the general direction of the field is trying to break through the analysis of bone density by AI on image pictures as a way to predict and diagnose osteoporosis more accurately.

**Table 5:** Top 20 keywords in the field of artificial intelligence in osteoporosis.

Rank	Keywords	Occurrences
1	Osteoporosis	231
2	Deep learning	149
3	Artificial intelligence	115
4	Bone mineral density	104
5	Diagnosis	70
6	Fracture	63
7	Machine leaning	62
8	Risk	51
9	Classification	43
10	Women	36
11	Neural network	35
12	Convolutional neural network	31
13	Prediction	29
14	Computed tomography	29
15	DXA	26
16	CT	25
17	Men	25
18	Management	24
19	Fracture risk	22
20	Postmenopausal women	22

In the keyword emergence analysis, the keyword “neural networks” exhibited the strongest burst intensity (3.85) during 2021–2022, signaling technological pivots. “Cancellous bone” showed the longest burst duration (2006–2020), reflecting sustained interest in trabecular microarchitecture. In addition, Recent emerging terms (2021–2024) include performance, vertebral fractures, bone diseases, convolutional neural network, microstructure, quantification, QCT, clinical use.

The technological advancements post-2020 have shown multidimensional breakthroughs: (1) The emergence of “performance” and “clinical use” reflects a shift in research focus toward validating the clinical efficacy of models. (2) The rise of “QCT” further emphasizes the demands for multimodal data integration. (3) Terms “microstructure” and “quantification” point to a trend toward more refined quantitative analysis of bone tissue.

As illustrated in Figure 7B, the field evolved through three phases: (1) Microarchitectural Exploration Phase (2006–2020): Centered on cancellous bone biomechanics. (2) AI Integration Phase (2018–2022): Marked by the combination of deep learning and imaging histology. (3) Clinical Translation Phase (2020-present): Focused on clinical validation and multimodal frameworks. This evolution from microstructural exploration to clinical translation marks a critical maturation of the field. The early stage was characterized by foundational science, establishing the link between bone structure and strength. The AI integration phase represents a methodological revolution where new tools enabled the analysis of previously unquantifiable features. The current clinical translation phase is most consequential, demonstrating a collective effort within the field to validate AI’s utility in real-world settings. The emergence of terms such as “performance” and “clinical use” reflects a growing imperative to meet the evidentiary standards required for regulatory approval and clinical adoption, moving beyond mere technical feasibility.

## 4 Discussion

### 4.1 General overview

The pressure to treat osteoporosis is increasing globally, which is strongly related to global aging, and in this context, the intervention of artificial intelligence technologies is particularly crucial. Using relevant visualization software, we analyzed the literature in this field for research progress and related hotspots from 2004 to 2024. The sustained growth in literature volume since 2019, culminating in an exponential surge of publications in 2024, marks a critical transition: AI applications in osteoporosis are evolving from the proof-of-concept phase to an intensive development and validation stage. This trend is likely driven by advances in deep learning architectures and growing recognition of their clinical potential.

Geographically, the research landscape is influenced by global imbalances in technological infrastructure and research funding. The dominance of North America, East Asia, and Europe indicates that AI-driven osteoporosis research remains primarily led by economically developed nations, highlighting the correlation between technological innovation and resource availability. While China and the United States lead in both publication volume and citation counts, the relatively weak direct collaboration between the two suggests that geopolitical and strategic factors may hinder optimal knowledge exchange. In contrast, the prominent role of the Technical University of Munich as a collaborative hub, along with its high total link strength, reflects the synergistic effects fostered by European integrated research policies that actively promote cross-border cooperation. This indicates that an institution’s centrality in the global network is not solely a function of output but is also significantly shaped by regional research ecosystems and policies. Furthermore, the highly cited journals are predominantly in the field of bone disease research (e.g., Osteoporosis International), indicating that the knowledge base of this domain remains closely aligned with traditional osteoporosis studies. While this ensures the clinical relevance of research outcomes, it may also introduce certain limitations. For instance, novel methodologies from core computer science or top-tier AI conferences may not be adequately integrated, which could, to some extent, constrain algorithmic innovation in the field.

### 4.2 Hotspots and frontiers

By analyzing the highly cited literature, co-cited literature and co-occurring keywords, research over the past decade has primarily focused on applying deep learning (DL) and convolutional neural networks (CNNs) to improve the diagnosis of osteoporosis through medical imaging, with the core being bone mineral density (BMD) assessment. This trend reflects the great potential of AI technology to enhance the early diagnosis of the disease.

Osteoporosis is an insidious disease with a low rate of early diagnosis, with only 15–30% of patients receiving a clinical diagnosis in the early stages of the disease (28). Because osteoporosis is difficult to detect in most patients before a fracture occurs, clinical strategies prioritize primary fracture prevention through early screening and intervention.

Currently, the diagnosis of osteoporosis primarily depends on quantitative patient-friendliness assessments of bone tissue structure, which can be clinically diagnosed in different body parts such as the spine, hip, limbs, etc. The techniques used for diagnosing osteoporosis mainly include DXA, QCT (29–32), and quantitative ultrasound (QUS) (33–35). DXA is the most widely available technique for measuring bone mineral content and can assess any skeletal region. The worldwide recognized diagnostic criteria for osteoporosis are a BMD T-score of ≤ − 2.5, with low bone mass (osteopenia) defined as a BMD T-score between −1 and −2.5 (1, 36, 37). Nevertheless, despite the advantages of DXA such as low cost and low radiation dose, its limitations are significant for the following reasons: (a) low diagnostic accuracy (38), (b) Diagnostic accuracy may be confounded by variables including race, geographic region, age, sex, anthropometric measures (height, weight, BMI), genetic predisposition, fracture history, lifestyle factors (diet, physical activity), medication use, and comorbidities (39, 40). (c) Reimbursement policy shifts. For instance, revisions to U.S. healthcare reimbursement policies have led many hospitals to reduce DXA utilization, thereby limiting the availability of analyzable DXA data (41, 42). (d) DXA, as a two-dimensional projection technique, is susceptible to adipose tissue interference, provides limited visualization of structural abnormalities, and lacks the capability for multidimensional analysis of bone geometry, size, and microstructure compared to QCT (43, 44). In contrast, QCT, though highly accurate, is costly and exposes patients to high radiation doses, while QUS, despite its portability and affordability, has limited utility in primary care setting (45). Additionally, existing clinical risk assessment tools (e.g., the Fracture Risk Assessment Tool (FRAX), the Q Fracture algorithm, the Garvan Fracture Risk Calculator, and the Osteoporosis Self-Assessment Tool) (46) can predict fracture risk based on known factors but fail to address the need for efficient for clinical purposes.

It is precisely these diagnostic challenges that have shaped the current research hotspots. The strong focus on “deep learning,” “CNNs,” and “diagnosis” in the literature indicates that the field is actively developing AI-based solutions to overcome the limitations of traditional methods. Therefore, the concentration on AI-driven image analysis can be understood as a direct effort to bridge the gap between clinical needs and the shortcomings of existing diagnostic technologies. Future progress is expected to focus on optimizing these models to improve their accuracy, generalizability, and integration into clinical workflows, thereby addressing the urgent need for early and efficient diagnosis.

### 4.3 Hotspot-opportunistic screening and diagnosis of osteoporosis

The keywords “deep learning” and “machine learning” indicate the wide range of applications and explorations of the AI technologies in the field of osteoporosis. The keywords “diagnosis,” “bone mineral density,” “prediction” and “risk” show the focus of applying AI to osteoporosis. The keywords “convolutional neural network,” “classification,” “management,” “DXA” and “CT” emphasize the role of AI in osteoporosis image analysis, processing, feature extraction, and classification. Combined with the content of high-impact publications, the current research hotspot centers on AI-driven diagnosis of osteoporosis through automated analysis of DXA, CT, and other imaging modalities. Furthermore, segmentation and recognition of thoracic, lumbar, or hip images serve as the cornerstone of osteoporosis screening, enabling early detection and risk stratification.

Deep convolutional neural networks (DCNNs) is one of the representative algorithms of deep learning, have achieved significant advancements in osteoporosis diagnosis. Its mechanism of operation is to process raw image pixels and associated class labels of the image data, automatically learn the feature representation with multiple levels of abstraction. This capability enables automated segmentation of 2D CT slices from routine health screenings and targeted diagnostic imaging (44). Experimental validation demonstrates (47) that chest radiograph-based deep learning models have the potential to be used for opportunistic automated screening for osteoporosis in a clinical setting. Imaging modalities that can be analyzed by DCNN are ultrasound, CT, MRI, and X-rays.

#### 4.3.1 DCNN-based X-ray imaging technology

In community hospitals with insufficient DXA machines, X-ray-based DCNN models offer a cost-effective and clinically feasible alternative for osteoporosis screening. Zhang B et al. developed a DCNN model to automatically classify lumbar spine orthostatic and lateral (L1–L2) X-ray images for normal BMD, osteoporosis, and osteopenia. The model achieved exceptional diagnostic performance (AUC = 0.990, sensitivity = 98%, specificity = 92.4%) in identifying osteoporosis, demonstrating high potential for opportunistic BMD screening in postmenopausal women. However, external validation and model optimization are required to enhance generalizability (48). Yamamoto et al. applied a CNN model to 1,131 hip X-ray scans, demonstrating high diagnostic accuracy (AUC = 0.92) for osteoporosis detection (49). Jang et al. developed a deep neural network (DNN) model based on VGG16 and a non-local neural network (NLNN) mode, pioneering the use of the Gradient Class Activation Mapping (Grad-CAM) to visualize model interpretability. The DNN achieved 81.2% overall accuracy, highlighting its utility as a clinical screening tool for osteoporosis risk prediction (50). Furthermore, Kavitha et al. combined a hybrid approach integrating histogram-based automatic clustering (HAC) algorithm and support vector machine (SVM) approach to predict postmenopausal osteoporosis via mandibular cortical bone analysis on dental panoramic radiographs (DPRs) (51).

#### 4.3.2 DCNN-based CT, QCT imaging technology

The contribution of Pickhardt PJ et al. in this area has been very significant. They have developed an automated algorithm and validated its feasibility for detecting cortical bone mineral density in CT scans (52, 53). In recent years, Pickhardt PJ et al. have conducted more in-depth researches in this area. For example, building on previous research, Pickhardt PJ et al. in 2020 utilized automated bone, muscle, and fat imaging biomarkers in abdominal CT images to predict fractures in terms of osteoporosis as opposed to the FRAX (54).

In contrast to his previous assays (55, 56), Pickhardt PJ developed a CT-based deep learning tool for automated assessment of BMD (57). His study demonstrated that the new DL tool had a higher success rate compared to previous tools in a heterogeneous abdominal CT cohort study. It simulates the placement of the region of interest (ROI) method of manually placing L1 trabeculae, outperforming previous feature-based algorithms in success rate and demonstrating greater adaptability to clinically diverse and non-standardized images compared to manual methods. Additionally, the tool detects vertebral compression fractures, reduces false-positive rates, and eliminates the need for additional radiation exposure, time, or costs (32, 54, 56–58). By integrating deep learning with traditional diagnostic models, future systems could further incorporate factors such as hip fracture risk and injurious fall assessment, thereby establishing a more clinically adaptive screening framework. This tool enables large-scale opportunistic osteoporosis screening via routine CT examinations, enhancing detection rates and enabling early clinical interventions.

Notably, several of the studies on osteoporosis have employed a fully convolutional neural network, U-Net, for automatic image segmentation of image pictures. For example, Liu et al. proposed an improved osteoporosis diagnostic algorithm based on U-Net network in 2020. Experimental results demonstrated that this method resolved image artifacts in bone density measurement while achieving a diagnostic accuracy of 81%, outperforming existing comparative approaches (59). For example, Fang et al. applied the full convolutional neural network U-Net to automatically segment spine and abdominal CT scan data from 1,499 patients, and then utilized the regression network DenseNet-121 for BMD calculation (60). The experimental results show that there is a strong correlation between automatically segmented BMD (via regression networks) and QCT-derived BMD. The model matched QCT in osteoporosis diagnostic accuracy while surpassing it in vertebral localization and segmentation precision. For example, Tang et al. utilized Mark-Segmentation-Net (MS-Net) for segmentation and then ROI to quantify the BMD (61). The results showed that the classification accuracy of BMD-Classification-Net (BMDC-Net) were 80.57, 66.21 and 82.55% for normal bone mass, low bone mass and osteoporosis, respectively. BMDC-Net outperformed DenseNet-121 in both accuracy and AUC, validating the efficacy of reducing network training parameters. Additionally, a visual diagnostic chart was generated to classify bone mass levels, assisting clinicians in formulating personalized follow-up strategies.

In recent years, multimodal integration for screening, diagnosis, classification, and prognostic assessment of osteoporosis has played a pivotal role. For instance, Zhang et al. in 2023 proposed a multi-task joint learning framework that transforms osteoporosis diagnosis from a binary to a ternary classification problem. By integrating localization, segmentation, and classification tasks, this framework addresses challenges such as high-dimensional data, multimodality, and multi-class imbalance, improving diagnostic accuracy (62). The proposed joint learning framework elevated classification accuracy from 82.1 to 93.3% in osseous tissue imaging analysis, validating its efficacy for enhancing diagnostic precision in osteoporosis assessment through multimodal feature fusion. Since the results of the study change with the normal or low dose data selected by the researchers, Zhang et al. also proposed a new multi-domain diagnostic and QCT image method called Deepmd QCT in 2024 (63). This is a multidomain diagnostic model that integrates different dosage images by analyzing different image domains to extracts shared features among them and estimating the gold standard QCT values from the CT values. Experimental results demonstrated a mean classification accuracy of 91% across dose domains and a goodness-of-fit of 0.95 with QCT. Its advantages include: (1) resolving diagnosis discrepancies caused by dose variations, (2) streamlined workflows with improved efficiency, and (3) reduced patient radiation exposure and healthcare costs.

AI has also contributed in the diagnosis of Osteoporotic vertebral fractures (OVFs) that are missed and underreported on CT examinations due to early asymptomatic conditions. Automated detection systems can reduce screening time and labor burden and improve diagnosis of asymptomatic early cone fractures.

For example, Tomita et al. developed a deep neural network-based automated detection system to detect unexpected OVFs in chest, abdominal and pelvic CT examinations (64). The system utilizes CNNs to extract radiological features and then aggregates the features via a sequence classifier [Long Short Memory Network (LSTM)] to predict the presence of vertebral fractures on CT scans. The experimental results showed that the accuracy and F1 scores on the independent test set are comparable to the diagnoses made by clinical imagers. Similarly, Bar et al. established a system with CT images to identify high-risk individuals for vertebral compression fractures (VCFs) and achieved an accuracy of 89.1%. However, this system could only detect the presence of vertebral fractures and deformities and could not differentiate between fresh and old fractures (65).

#### 4.3.3 DCNN-based MRI imaging technology

Early diagnosis and treatment of fresh osteoporotic vertebral fractures (OVFs) are clinically meaningful, but it is challenging due to the lack of morphological changes in occult OVFs on radiographs and the difficulty in accurately assessing the degree of fracture freshness on CT examination. In recent years, researchers have used AI to analyze fresh OVFs on MRI images, which can improve the accuracy of their imaging diagnosis. For example, Yabu et al. in 2021 developed an automated system for detecting fresh OVFs on MRI images using CNN (66). The diagnostic accuracy of CNN and two spine surgeons for 100 cones were: 88.0%/87.0%/80.0%, respectively. The results showed that the system’s performance is comparable to that of an experienced spine surgeon and it can help clinicians diagnose OVFs. In another study, Yoda et al. employed a CNN model to differentiate osteoporotic vertebral fractures (OVFs) from malignant vertebral compression fractures (MVFs) on MRI, surpassing spine surgeons in diagnostic accuracy (67).

Automated systems can significantly alleviate the unequal distribution of healthcare resources, especially in primary care hospitals, and enable efficient, standardized osteoporosis screening by reducing reliance on high-cost equipment and specialists. Their low-cost and easy-to-deploy nature enhances early detection of osteoporosis and fractures in underserved regions, facilitating timely clinical interventions.

### 4.4 Challenges of artificial intelligence in osteoporosis

Despite the considerable potential of artificial intelligence as an intelligent engine and a catalyst for advancements in osteoporosis treatment, its path from research and development to clinical application faces multiple challenges that require in-depth examination. A more profound analysis reveals that these challenges not only pertain to technical performance but also fundamentally impact clinical translation, regulatory approval, and ethical deployment.

The scarcity of high-quality, large-scale, and diverse datasets remains a major bottleneck. Many models in current research rely on retrospective, single-center data, which inevitably introduces data bias. A typical example is that a fracture risk prediction model trained primarily on data from postmenopausal women in Western Europe may exhibit significantly reduced performance when applied to Asian male patients or younger individuals. Such bias not only threatens the general applicability of the models but also risks exacerbating existing healthcare disparities, leading to underdiagnosis or overdiagnosis in underrepresented populations (48, 68). To address this issue, future research must prioritize prospective, multi-center collaborations to establish meticulously annotated datasets encompassing diverse ethnicities, genders, ages, and comorbidities. Furthermore, the adoption of privacy-preserving techniques such as federated learning can enable cross-institutional model training without sharing raw patient data, thereby effectively enhancing data diversity while ensuring data security.

The “black box” nature of many complex AI models poses a fundamental obstacle to clinical translation. The opacity of their decision-making processes—for instance, when predicting a patient’s 10-year fracture risk—makes it difficult for clinicians to comprehend and trust the output, leading to reluctance in incorporating it into critical treatment decisions. This lack of interpretability not only erodes the doctor-patient relationship but also creates a “translation gap” between research and clinical practice: a model demonstrating high accuracy on retrospective datasets may never be utilized in real-world clinical settings due to clinicians’ distrust. To bridge this gap, the field can develop “Explainable AI (XAI)” methods, such as generating saliency maps that highlight critical imaging features or providing simplified decision rules (69, 70). These tools can transform AI from a “black box” into a “transparent advisor” understandable to clinicians, which is essential for building trust, ensuring accountability, and achieving meaningful clinical integration.

The issue of insufficient generalization ability in AI models is directly related to their clinical safety. A model may perform excellently on its training dataset, but due to overfitting and the significant differences in real-world patient populations, imaging protocols, and disease presentations, its performance may decline sharply during external validation or clinical deployment (68). For example, a volumetric bone mineral density measurement model trained solely on lumbar spine QCT data may not accurately assess hip bone mineral density, as the bone structure and imaging characteristics of these two sites differ. This highlights the critical importance of rigorous external validation on diverse datasets (48, 56). Solutions include adopting more robust algorithms and ultimately demonstrating through large-scale, prospective clinical trials whether AI tools can produce measurable impacts on improving patient outcomes (such as reducing actual fracture incidence rates), rather than merely enhancing diagnostic accuracy.

Beyond technical and operational challenges, ethical and privacy concerns constitute fundamental barriers to the clinical deployment of artificial intelligence. A typical dilemma is that developing AI models requires sharing large amounts of sensitive patient data, which conflicts with the core obligation to protect patient privacy. If a dataset used for predicting fracture risk is leaked during sharing or storage, it could lead to the misuse of sensitive information such as patients’ health status, severely compromising patient trust (70, 71). A more profound impact is that such concerns may hinder data collaboration between healthcare institutions and research entities, fundamentally slowing the progress of the entire field. Furthermore, algorithmic bias is not merely a technical issue but also a serious ethical concern. If AI models are primarily trained on data from specific populations (e.g., urban high-income groups), they may underperform when serving rural or economically disadvantaged groups, thereby exacerbating existing healthcare disparities (72, 73). The solutions must be systematic: at the technical level, privacy-enhancing technologies such as federated learning should be adopted; at the governance level, strict and transparent data governance frameworks must be established to clarify data ownership, informed consent, and access rights; finally, industry and regulatory bodies should jointly develop ethical review guidelines tailored to osteoporosis AI applications, incorporating fairness audits and bias mitigation as essential steps in model validation.

Ultimately, transforming AI into reliable clinical tools requires addressing the core challenge of establishing clear regulatory and legal liability frameworks. Ambiguous approval pathways and potential liability risks can significantly hinder innovation and clinical adoption. Therefore, future efforts must focus on cross-disciplinary systemic actions. Concurrently, clear guidance should be provided for different stakeholders: researchers and developers should prioritize creating interpretable models that meet regulatory standards and actively seek prospective clinical trials to demonstrate their clinical utility; clinicians and hospitals should participate in the co-design and clinical validation of these tools and develop protocols to clarify liability norms for AI-assisted decision-making; regulatory bodies and policymakers must urgently establish adaptive approval pathways and ethical guidelines tailored to the dynamic nature of AI to safeguard safe and effective innovation.

In summary, challenges such as data bias, the black-box nature of models, ethical dilemmas, and regulatory ambiguity do not exist in isolation but are interconnected, collectively forming a “translational gap” that hinders the progression of AI from research to widespread clinical application. Bridging this gap requires far more than algorithmic optimization; it urgently demands a fundamental paradigm shift—from a purely technology-driven approach to building an ecosystem that integrates interdisciplinary collaboration, clinical validation, and robust governance frameworks. Only through such systemic efforts can the transformative potential of AI in the field of osteoporosis be fully unlocked.

## 5 Conclusion

This study represents the first comprehensive bibliometric analysis of AI developments in the osteoporosis field. The rapid evolution of this domain stems from the global aging population’s demand to overcome the limitations of traditional diagnostic methods like DXA. Current research is distinctly focused on AI-driven medical imaging, particularly utilizing deep learning to automatically analyze X-ray, CT, and MRI data, indicating a transition from technological development toward clinical translation.

Based on our analysis, we propose the following strategic directions: For researchers: Future work should move beyond pure algorithmic performance. It is essential to prioritize improving model generalizability across diverse populations and imaging protocols, develop reliable explainable AI (XAI) techniques to build clinical trust, and conduct rigorous prospective validation studies to demonstrate tangible clinical utility. For clinicians and policymakers: Engaging in the co-design of AI tools is crucial to ensure they integrate into clinical workflows and address practical needs. Furthermore, there is an urgent need to develop adaptive regulatory frameworks and reimbursement policies that can keep pace with the iterative nature of AI-based software, thereby facilitating their responsible and equitable adoption.

We acknowledge the limitations of this study. First, relying solely on the Web of Science Core Collection, while ensuring data quality and compatibility, may have omitted relevant studies from other databases (such as Scopus or PubMed), potentially introducing selection bias. Second, restricting the analysis to English-language articles may have overlooked significant contributions in other languages. Third, although sensitivity checks were performed, the parameters of the bibliometric software can influence network visualization and clustering outcomes. Future bibliometric research could benefit from integrating multiple databases, adopting more advanced techniques to explore thematic evolution in depth, and conducting comparative analyses of AI applications across different disease areas (such as cardiology or oncology), thereby extending this methodology to other AI-related medical fields.

In summary, fully realizing the transformative potential of AI in the field of osteoporosis requires collaborative efforts focused on developing robust, clinically relevant, and transparently integrated solutions.

## Data Availability

The original contributions presented in the study are included in the article/supplementary material, further inquiries can be directed to the corresponding authors.
